# Supplementary material for: Augmentation Method for High Intra-Class Variation Data in Apple Detection
Source: Sensors (Basel). 2022 Aug 23;22(17):6325. doi: 10.3390/s22176325 (PMC9460715; doi:10.3390/s22176325)
Supplement: Supplementary file 1 [file sensors-22-06325-s001.zip › sensors-1845275-supplementary.pdf]

sensors

MDPI

Augmentation method for high intra-class variation data in apple detection （Supplementary Materials）

Huibin Li[1], Wei Guo[2*], Guowen Lu[3], Yun Shi[1*]

[1] Institute of Agricultural Resources and Regional Planning, Chinese Academy of Agricultural Sciences, Beijing, China
[2] Graduate School of Agricultural and Life Sciences, The University of Tokyo, Tokyo, Japan
[3] College of Urban and Environmental Sciences, Central China Normal University, Wuhan, China
* Correspondence: shiyun@caas.cn (Y.S.); guowei@g.ecc.u-tokyo.ac.jp (W.G.)

Abstract: Deep learning is widely used in modern orchard production for various inspection missions, which helps improve the efficiency of orchard operations. In the mission of visual detection during fruit picking, most current lightweight detection models are not yet effective enough to detect multi-type occlusion targets, severely affecting automated fruit-picking efficiency. This study addresses this problem by proposing the pioneering design of a multi-type occlusion apple dataset and an augmentation method of data balance. We divided apple occlusion into eight types and used the proposed method to balance the number of annotation boxes for multi-type occlusion apple targets. Finally, a validation experiment was carried out using five popular lightweight object detection models: yolox-s, yolov5-s, yolov4-s, yolov3-tiny, and efficidentdet-d0. The results show that, using the proposed augmentation method, the average detection precision of the five popular lightweight object detection models improved significantly. Specifically, the precision increased from 0.894 to 0.974, recall increased from 0.845 to 0.972, and mAP0.5 increased from 0.982 to 0.919 for yolox-s. This implies that the proposed augmentation method shows great potential for different fruit detection missions in future orchard applications.

Keywords: deep learning; modern orchards; visual detection; fruit picking; lightweight detection models; augmentation method

Citation: Li, H.; Guo, W.; Lu, G.; Shi, Y. Augmentation Method for High Intra-Class Variation Data in Apple Detection. *Sensors* **2022**, *22*, 6325. https://doi.org/10.3390/s22176325

Academic Editor: Yongwha Chung

Received: 17 July 2022
Accepted: 18 August 2022
Published: 23 August 2022

Publisher's Note: MDPI stays neutral with regard to jurisdictional claims in published maps and institutional affiliations.

Copyright: © 2022 by the authors. Licensee MDPI, Basel, Switzerland. This article is an open access article distributed under the terms and conditions of the Creative Commons Attribution (CC BY) license (https://creativecommons.org/licenses/by/4.0/).

1. Method 1

1. BL synthesis method: First, calculate the number of BL to be synthesized. Then, use L synthesis method to synthesize the L image. Subsequently, L image will be used as the basis. Randomly obtain the branch elements from the component pool according to illumination demand. Then, randomly fill branches on the L image; refer to B synthesis method. Finally, obtain the BL image.
2. BF synthesis method: First, calculate the number of BF to be synthesized. Second, use the F synthesis method to synthesize F image. Then, use F image as the basis, and obtain the branch elements from component pool according to illumination demand. Subsequently, randomly fill the branches on the F image; refer to the B synthesis method. Finally, obtain the BF image.
3. LF synthesis method: First, calculate the number of LF to be synthesized. Then, use the F synthesis method to synthesize the F image. Next, randomly obtain the leaf elements from the component pool according to illumination demand. Then, the leaf image is randomly filled on the F image referring to the L synthesis method. Finally, the LF image will be obtained.
4. BLF synthesis method: First, calculate the number of BLF to be synthesized. One method is to synthesize the LF images according to the LF generation method. Next, obtain random branch elements from the component pool according to illumination demand. Then, the branch element is randomly filled on LF image with reference to B synthesis method. Finally, the BLF image will be obtained. Another method is to

extract a composite occlusion element first. Next, resize the element to the size of N. Finally, the resized element is attached directly to N. Then, the BLF image will be obtained.

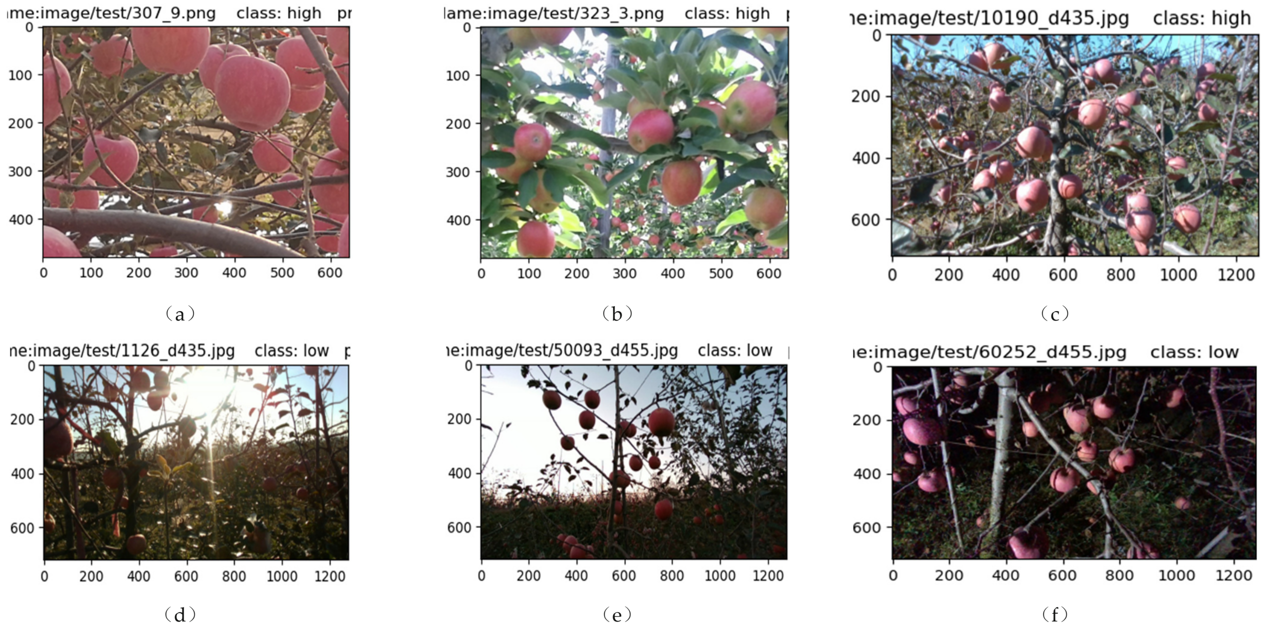

Figure S1. Results of raw images classified by illumination: (a) The image illumination classification result of QX_H is high. (b) The image illumination classification result of PSR_H is high. (c) The image illumination classification result of ZY_H is high. (d) The image illumination classification result for ZY_L is low. (e) The image illumination classification result of ZY_L is low. (f) The image illumination classification result of ZY_L is low.

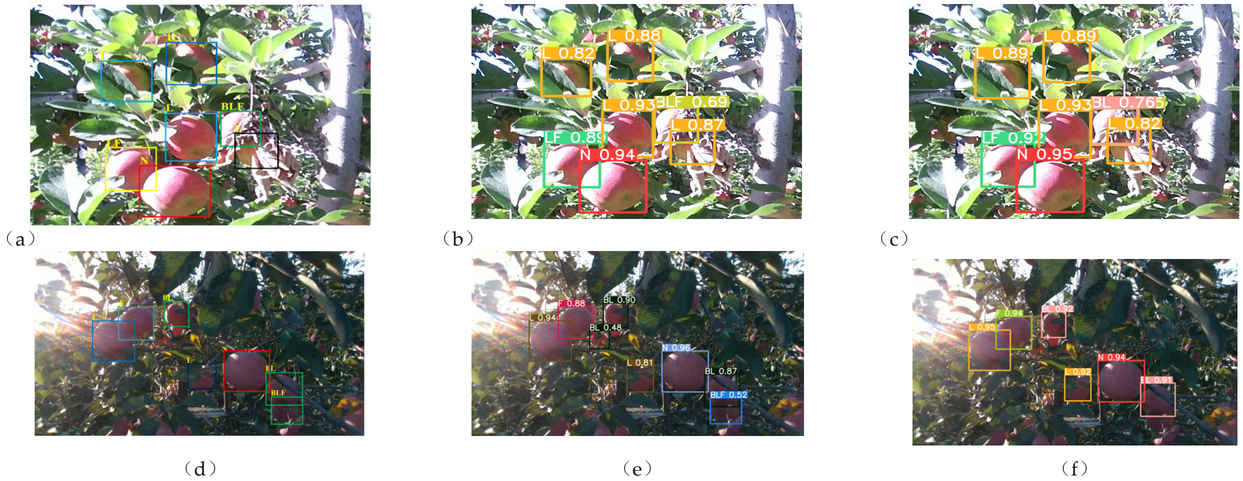

Figure S2. Visualization test results of yolov5-s-basic and yolov5-s-bal: (a) Annotated image within PSR_H_TEST. (b) Figure (a) result after being tested by the yolov5-s-basic model. (c) Figure (a) result after being tested by the yolov5-s-bal model. (d) Annotated images within ZY_L_TEST. (e) Figure (d) result after being tested by the yolov5-s-basic model. (f) Figure (d) result after being tested by the yolov5-s-bal model. Red circles represent incorrect test results; yellow circles represent correct test results.

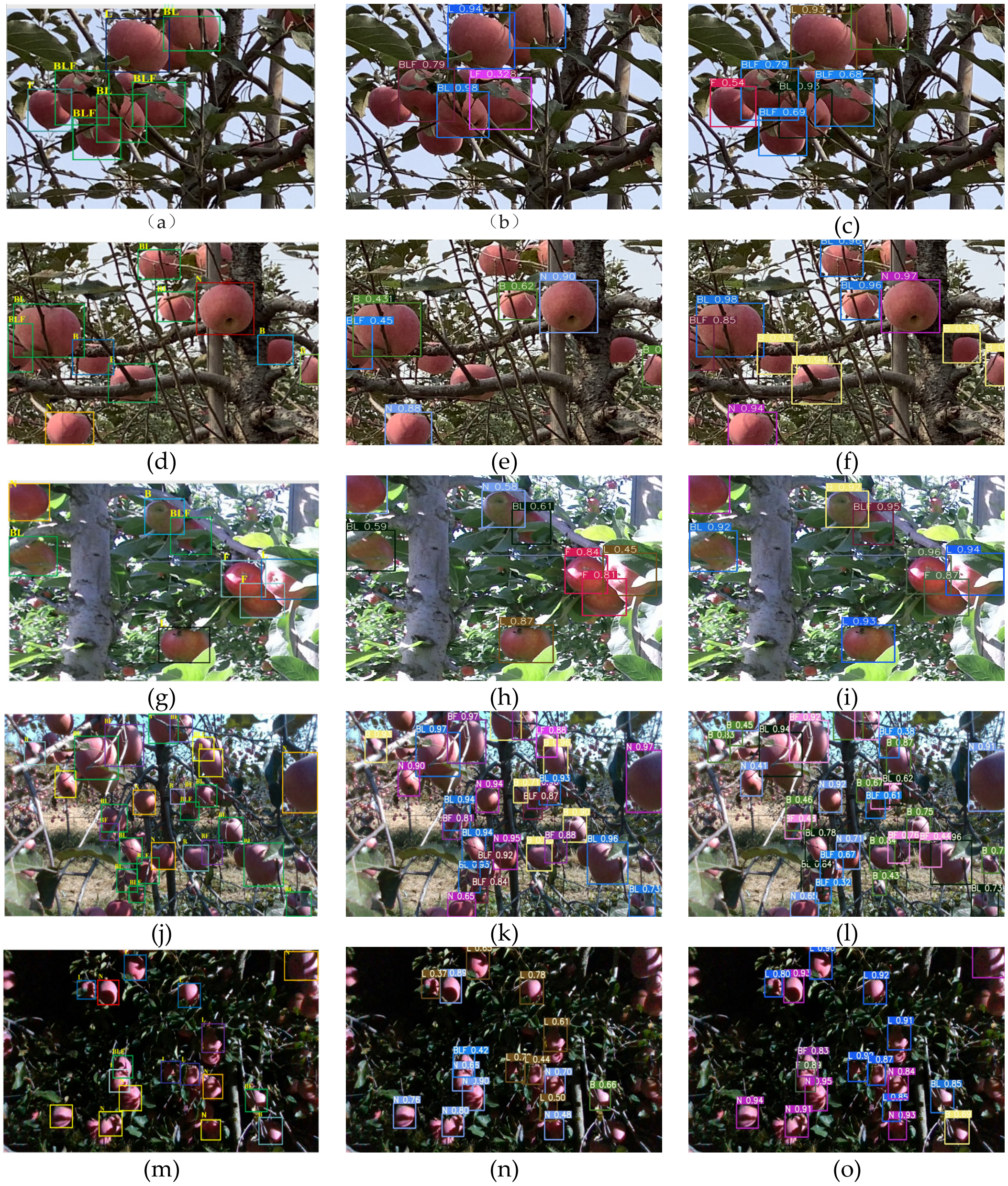

Figure S3. Visualization test results of yolov4-s-basic and yolov4-s-bal: (a) Annotated image in QX_H_TEST. (b) Figure (a) result after being tested by the yolov4-s-basic model. (c) Figure (a) result after being tested by the yolov4-s-basic model. (d) Annotated image in QX_H_TEST. (e) Figure (d) result after being tested by the yolov4-s-basic model. (f) Figure (d) result after being tested by the yolov4-s-basic model. (g) Annotated image in PSR_H_TEST. (h) Figure (g) result after being tested by the yolov4-s-basic model. (i) Figure (g) result after being tested by the yolov4-s-bal model. (j) Annotated image in ZY_H_TEST. (k) Figure (j) result after being tested by the yolov4-s-basic model. (l) Figure (j) result after being tested by the yolov4-s-bal model. (m) Annotated image in ZY_L_TEST. (n) Figure (m) result after being tested by the yolov4-s-basic model. (o) Figure (m) result after being tested by the yolov4-s-bal model. Red circles represent incorrect test results; yellow circles represent correct test results.
